# The mini-open procedure with a modified figure of eight for managing sacrococcygeal fracture-dislocation: A case report and literature review

**DOI:** 10.1016/j.ijscr.2024.109769

**Published:** 2024-05-16

**Authors:** Romy Deviandri, Bayu Pratama Putra Pribadi, Muhammad Wiranata

**Affiliations:** a Department of Surgery-Faculty of Medicine, Universitas Riau, Arifin Achmad Hospital, Pekanbaru, Indonesia; b Department of Orthopedics, University of Groningen, University Medical Center Groningen, Groningen, the Netherlands

**Keywords:** Fracture fixation, Suture techniques, Sacrum, Fracture dislocation, Case report

## Abstract

**Introduction and importance:**

A fracture associated with an anteriorly displaced fragment may induce soft tissue disintegration. However, this might be avoided by maintaining the stability of the sacrococcygeal bone. Fixation by using less invasive modalities is needed to improve the outcome.

**Case presentation:**

A 37-year-old female came with tailbone pain, which lasted around one month. There was a history of falling in a sitting position a month before hospital admission. Tenderness was positive while palpating the perineal site. A radiography examination shows a fracture in the sacrococcygeal segment with anterior dislocation. The patient was diagnosed with a sacrococcygeal fracture and anterior dislocation.

**Clinical discussion:**

We performed a mini-open procedure using a modified figure-of-eight technique to reconnect the sacrococcygeal bone. Suturing was performed through the skin in the painful area, and then the bone at the injured site was reduced. As an outcome, there was an improvement in the Visual Analogue Scale (VAS), Oswestry Disability Index (ODI), and EuroQol-5 Dimensions (EQ5D) scores.

**Conclusion:**

A mini-open procedure with a modified figure of eight is a simple and valuable method for correcting the sacrococcygeal components.

## Highlights

• Sacrum and coccygeal fracture-dislocation was an uncommon accident and frequently misdiagnostic
• Injuries with a risk of reducing the quality of a patient's life should receive priority treatment.
• In this condition, mini-open techniques using figure-of-eight surgical procedures are safe and viable options.
• A good improvement is achieved based on the VAS, ODI, and EQ5D scales.

## 1 Introduction

The coccyx—sometimes called the tailbone—is the last bone at the end of the spine. The stability of the sacrococcygeal region results from the bone structure associated with the integrity of surrounding tissues. Instability will cause coccyx pain, usually caused by a contusion and fracture in the sacrococcygeal area. The exact prevalence data for sacrococcygeal fractures is not known. Thus, the effect of seasonality and weather could be the risk factor for accidents [1]. Although many cases are self-limiting, some require surgery to reduce the risk of malformation. The situations are challenging to treat, mainly when associated with other comorbidities [2]. Clinicians should understand the variety of diagnostic modalities for managing coccyx pain. These situations could increase the patient's quality of life [3].

Fixation with surgery is an option for restoring normal function in the case of a coccyx fracture-dislocation. Additionally, persistent instability of the dislocated joint could cause prolonged coccydynia [4]. A conservative treatment and less invasive procedure by close reduction could be used in the acute phase. Nevertheless, an open procedure may be considered if the closed reduction procedure fails [5].

This case report will discuss a fracture in the sacrococcygeal segment associated with anterior dislocation, managed by a mini-open procedure using a figure-of-eight suturing technique, and evaluate the outcome after treatment. This case was reported based on the guidelines of SCARE 2023 [6].

## 2 Case presentation

A 37-year-old female was taken to the hospital with chief complaints of aggravating tailbone pain, rating it an 8–9 on the Visual Analogue Scale (VAS), which she felt for around one month. The patient had a history of falling in a sitting position with formidable contact in the buttocks area with the ground of about 1 m. The patient was not using walking assistance or allergy medicine for an extended period. There is no accommodation while working as a household servant.

**Fig. 1:**
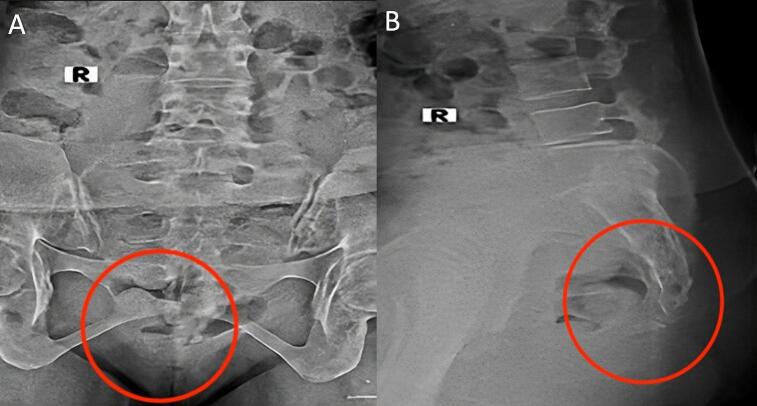
The X-ray examination before surgery showed a sacrococcygeal fracture in AP view (a) and lateral view (b).

Swelling with deformity was found in a physical examination of the buttocks area. Pain and tenderness were complained of during palpation. The pain was aggravated, especially in a sitting position. Radiological assessment of the sacrococcygeal bone showed a discontinuity in the sacrococcygeal bone. Based on physical and radiology examinations, the patient was diagnosed with a sacrococcygeal fracture-dislocation (Fig. 1).

**Fig. 2:**
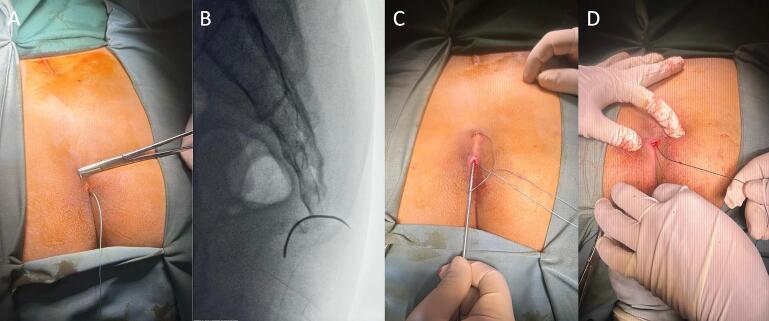
The patients under general anaesthesia are in the prone position. A minimal incision of about 1 cm was performed close below the fracture site; then, the initial suture was passed. (a) A lateral X-ray view showed a needle connecting the sacrum and coccygeal using intraosseous procedures. (b) After the first suture, the thread was pulled back into the initial incision subcutaneously. (c) A minimal invasive figure of eight procedures was figured out, and the suture's end was tied (d).

Previously, two weeks before admission to the hospital, the patient had received conservative therapy by administering non-steroid anti-inflammatory drugs, diclofenac sodium 50 mg two times daily and a special cushion for the buttock area. However, there was no improvement in the patient's quality of life. Subsequently, we chose a close reduction procedure that collaborated with a pain management procedure by a caudal epidural steroid injection with 1 cm^3^ dexamethasone and lidocaine 0,5 %. Still, the patient complained about pain, and we found instability. One week later, after discussing with the patient, we considered performing a fixation with a mini-open procedure. It was performed to fix the fracture fragment and prevent soft tissue injury. The procedure was performed in November 2023. These surgical techniques are listed below (Fig. 2).

**Fig. 3:**
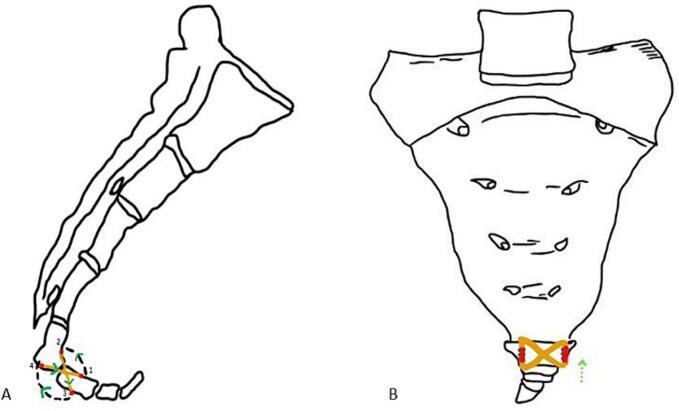
Illustration of a Two-dimensional sacrococcygeal fracture with mini-open reduction and fixation by a modified figure of eight suture lateral view (a) and AP view (b).

A modified figure of eight sutures was performed to immobilize each segment and fix the sacrum and coccygeal structure using a non-absorbable thread (Ethibond Excel, 2-0, Ethicon). After surgery, the patient's hemodynamics improved, and there was no need for intensive care at the surgical intensive care Unit (ICU). The patient was taken to the inpatient room for more observation and was given ketorolac as an analgesic three times daily intravenously. The buttocks segment of the patient was shielded with gauze; excessive pressure in the buttocks area should be avoided (Fig. 3).

**Fig. 4:**
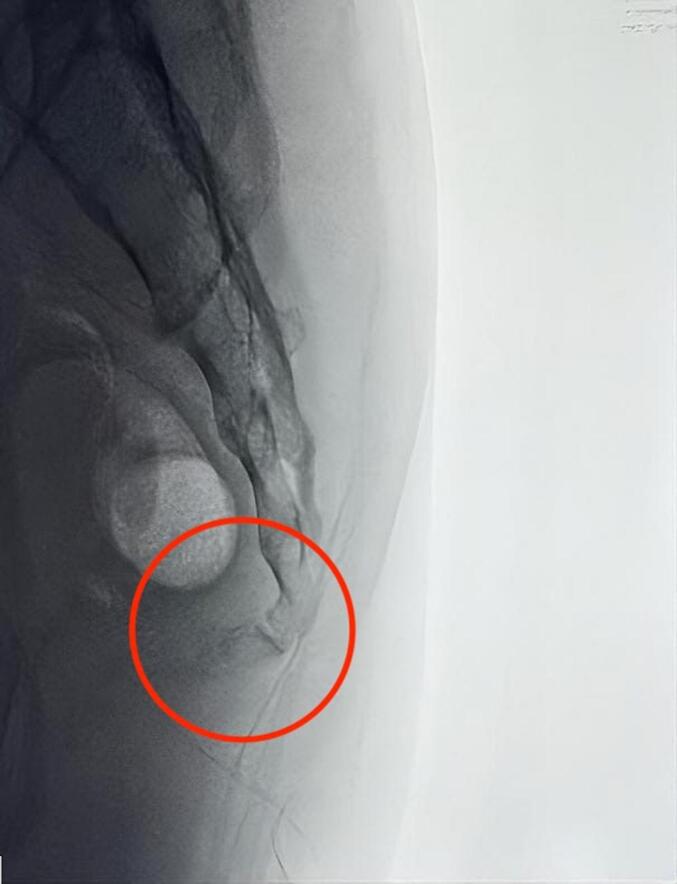
Lateral view sacrococcygeal X-ray at three months postoperative.

Next, the patient was brought to physical therapy to improve ROM and relieve pain as tolerated. No significant limitations were found in the rehabilitation phase. The clinician advises avoiding complex manoeuvres such as lifting or carrying something heavier after surgery and performing gradual rehabilitation as pain is tolerated. Six weeks after surgery, the patient was able to perform daily activities such as sitting and even washing his laundry alone. Nevertheless, the minimal pain was still found and localized predominantly in the surgical area while gaining heavy property with VAS 1–2. The x-ray showed an acceptable and stable sacrococcygeal bone three months after surgery. A good outcome was achieved based on the Oswestry Disability Index (ODI) score, which decreased from 16 to 3, and the EuroQol-5 Dimensions (EQ5D) score increased from 0,51 to 0,92 (Fig. 4).

## 3 Discussion

This case report aims to examine the improvement results of a patient treated with the mini-open procedure with a modified figure of eight suturing techniques for sacrococcygeal fracture-dislocation. Three months after surgery, acceptable results were obtained from the procedures. A significant outcome is attained based on the VAS, ODI, and EQ5D scores.

Sacrococcygeal fracture with dislocation is a rare case. This is usually the manifestation of traumatic situations. No specific interventions are available to manage the fracture dislocation; most therapeutic interventions do not perfectly realign the fractured site [7]. In the acute phase, conservative therapy is initiated, such as an analgetic drug, using stool softeners to decrease coccyx pain during bowel movement, and a special cushion for the buttock area. Although most patients show pain relief with conservative treatment, some cases show persistent coccyx pain [8]. This condition is called coccydynia and is characterized by pain surrounding the buttocks area, which worsens when performing high-pressure movements on hard surfaces. A reduction should be considered to manage further coccyx pain instability related to conservative treatment. Also, women should be aware of sexual activities and childbirth after these conditions happen.

Closed reduction may be a method where the gloved finger is located through the anal, and the suturing coordinate is connected to the disengaged fragment of the coccyx joint, predominantly felt in the rectal mucosa. These strategies could be primary reduction procedures within the acute phases; successful procedures may improve early recovery.

Williamson described a case including a 30-year-old female who had a sacrococcygeal fracture accompanied by an anterior dislocation. They anticipated using closed reduction to improve sexual activities. A closed reduction under fluoroscopy was performed related to young age. In this case, the coccygeal structure tends to move anteriorly, and after conducting a close reduction with local anaesthesia and relaxant, the close reduction was successful [5]. Significant pressure on the mucosal part of the rectal is required to achieve joint reduction; the risk of cortical breakage also increased after several manoeuvres of surgical intervention.

Some of the failed close reductions could be found [5]. In this situation, a fixation by open reduction procedure could be the option [9]. Verhaar et al. performed trans-osseous suturing while implementing the open reduction procedure. Procedure performed when open reduction could gain stability by a four-strand suturing after reduction bone [9]. A four-strand suture may require a larger incision, increasing the risk of infection. In contrast to our case, a closed reduction was performed to realign the sacrococcygeal using a minimally invasive method. The figure of eight sutures, conducted by the authors with a mini-open approach, required only a small incision. The modified suturing process was passed through the skin and fixed through the incision site to stabilize the close reduction procedure.

It makes sense to use conservative techniques for coccyx fracture-dislocation patients initially. Effective pain management techniques are necessary for improving the outcome. A caudal epidural block or a ganglion impar block could be the options [10]. However, a surgical method should be considered if the case progresses to prolonged coccyx pain. In our report, we were able to fix the fracture-dislocation and manage the pain while preventing complications associated with surgery performed using a mini-open procedure with a modified figure of eight suture method.

## 4 Conclusion

The mini-open procedure, which uses the modified figure of eight suturing technique, is an option to fix a sacrococcygeal fracture-dislocation. Improvement was demonstrated by reducing coccyx pain symptoms and improving VAS, ODI, and EQ5D scores.

## Informed consent

Written informed consent was obtained from the patient for publication of this case report and accompanying images. A copy of the written consent is available for review by the Editor-in-Chief of this journal on request

## Ethical approval

Our institution does not have any specific ethical requirements for the publication of case reports. Since this report pertains to a single case, it is exempt from ethical review in our hospital.

## Funding

This case report received no specific grant from public, commercial, or not-for-profit funding agencies.

## Author contribution

Study concept or design, data analysis or interpretation: Romy Deviandri.

Data Collector and writer: Bayu Pratama Putra Pribadi, Muhammad Wiranata.

## Guarantor

Romy Deviandri.

## Provenance and peer review

Externally peer-reviewed. Not commissioned.

## Conflict of interest statement

The authors affirm no conflict of interest in this study.
